# Online Flow Measurement of Liquid Metal Solutions Based on Impact Force Sequences: Modeling Analysis, Simulation, and Validation of Experimental Results

**DOI:** 10.3390/s24144553

**Published:** 2024-07-14

**Authors:** Qiguang Li, Xiru Zheng, Yu He, Fangmin Xu, Yulin Zhuang, Bingji Zeng, Bofang Duan

**Affiliations:** 1 School of Mechanical and Electrical Engineering, Beijing Information Science and Technology University, Beijing 100192, China; 2021020013@bistu.edu.cn (X.Z.); 2022019026@bistu.edu.cn (Y.H.); 2023020012@bistu.edu.cn (Y.Z.); 2021010281@bistu.edu.cn (B.Z.); 2022010111@bistu.edu.cn (B.D.); 2 Beijing University of Posts and Telecommunications, Beijing 100876, China; xufm@bupt.edu.cn

**Keywords:** impact force, metal melts, modeling analysis, validation of experimental results

## Abstract

Aiming at the existing high-temperature liquid metal flow online accurate measurement by the metal melt characteristics, installation space, and high-temperature environment adaptability limitations, this paper innovatively puts forward a soft measurement method based on the impact force generated in the fluid flow process as an observational variable series. Fluid mechanics theory and simulation software are used to analyze and verify the feasibility of the impact force as an observable variable to measure the flow rate, followed by the construction of the CNN-LSTM-CNN-Double (CLCD) flow measurement model of impact force and flow rate based on the parameters of the learning rate and the number of training times, and finally the construction of a test platform for the flow measurement, and the validity of the method is verified through actual operation.

## 1. Introduction

High-temperature liquid metal flow technology is a core driver for the continued development of the nuclear, metallurgical, and related industries. For example, high-temperature liquid metal is used as an ideal coolant in fields such as nuclear reactors and aerospace engines [1], and the precise acquisition of real-time flow and flow information is one of the key technologies to ensure safe control. In the casting industry, by accurately controlling the flow of liquid metal, not only can it effectively improve the quality of castings and prevent the production of defects such as rolled slag and billet cracks [2], but also realize the fine regulation of grain growth. Therefore, the flow measurement of high-temperature liquid metal solution is of great significance in promoting the progress of related industrial fields [3]. Currently, the flow control of high-temperature metal solutions in the industrial casting process mainly relies on the adjustment of various parameters by manual experience; however, in the face of liquid level fluctuations influenced by various factors, this method is difficult to maintain a stable flow rate. Metal solution, due to its high temperature, easy oxidation solidification, cooling solidification, ease to stick, and other characteristics, is injected into the mold cavity with rapid fluidity. At the same time, due to the harsh measurement environment and limited sensor installation space, conventional high-temperature liquid metal flow measurement techniques such as turbine flowmeter [4], orifice plate flowmeter [5], optical probe method [6], and Coriolis flowmeter [7] have limitations, mainly including limited practical applications, susceptibility to oxidation and corrosion, measurement space limitations, and the impact of external conditions; insufficient interference resistance and stability, especially in the noise environment, is prone to error; measurement accuracy is limited by the purity of the solution, flow rate and flow field complexity, and limited transmission and shooting area. Therefore, the online accurate flow measurement in the metal solution casting process is still a technical challenge. The accurate online measurement of solution flow in the industrial casting process for metal solutions is still a key issue to be solved.

The challenges of high-temperature liquid metal flow measurement technology have prompted researchers to explore soft measurement techniques. This technology solves spatial constraints and optimizes the operating conditions of high-temperature liquid metals by finding auxiliary variables related to flow and combining modern information processing techniques, such as deep learning models, to achieve online flow measurement. The selection of auxiliary variables in soft measurement methods is particularly important. It is necessary to choose process variables that have a strong correlation or causal relationship with the dominant variables, and follow the principles of sensitivity, specificity, and ease of access [8]. For example, scholars such as Jagad PI derived mass flow by measuring substrate stress [9]; Zhao YJ and others measured electrostatic induction charges using annular sensors, measured gas flow velocity using ultrasound, and derived smoke dust mass flow from friction current and gas velocity [10]; and Jaiswal SK and others used a weighing method to indirectly measure water flow [11]. Based on the actual industrial casting environment and metal solution characteristics, and drawing on previous researchers’ selection of auxiliary variables, three key variables related to outlet flow were identified: the impact force of the solution impacting the inclined plane, flow velocity, and mass. However, due to the fast flow velocity and easy oxidation of metal solutions, traditional methods of measuring flow through flow velocity or mass are difficult to achieve. Therefore, a soft measurement scheme for flow based on impact force is proposed: pressure sensors are installed at the bottom of the impact pipe at the outlet of the metal solution to collect the force of the solution impacting the inclined plane, thereby measuring the flow rate. This method takes into account the characteristics of metal solutions while satisfying the limiting conditions of the casting environment, making it practically operable.

The modeling of soft measurement technology is the core issue for achieving flow measurement, mainly including mechanism modeling and data-driven modeling. Mechanism modeling reveals the complex coupling relationship between parameters through physical or chemical equations [12], while data-driven modeling establishes the mapping relationship between auxiliary variables and dominant variables through autonomous learning. Researchers will combine these two methods [13] and strive to establish a precise correlation model between impact force and flow to promote the development of high-temperature liquid metal flow measurement technology. Since the relationship between impact force and flow is usually nonlinear, traditional methods may not accurately describe this relationship. The data-driven deep learning method has powerful feature extraction capabilities and can take into account the complex characteristics of the nonlinear, time-varying, and transient effects of the relationship between impact force and flow. The neural network based mainly on convolutional neural networks (CNN) and Long-Short Term Memory (LSTM) has more advantages in feature extraction and time series processing. CNN, with its convolutional layer as the core, can effectively extract sequence features and other data through convolutional operations, capturing local patterns and structures [14]. Wang et al. improved on the basis of CNN (convolutional neural network). They designed a temporal convolutional network to estimate liquid volume flow, and this innovative method significantly improved the accuracy of flow estimation [15]. At the same time, scholars such as Zhang improved the transient flow measurement method of gas–liquid two-phase flow by adjusting the parameters of the CNN network and utilizing its powerful feature extraction capabilities, achieving remarkable results [16]. LSTM performs well in processing time series data. Through its unique memory unit structure, it can better learn the relationship between the front and back characteristics [17] and capture long-term dependencies in fluid flow during flow measurement. Jin J et al. successfully used LSTM to analyze the vibration response of the metering plate, calculate the vibration level and roughness, and use them as input features. At the same time, they used the actual mass flow measured by the weighing sensor as the output feature of the LSTM prediction model. Experimental results showed that the deviation of their flow prediction was less than 6%, indicating the high accuracy of the model [18]. In addition, Yan, L et al. also used LSTM to conduct a deep analysis of past water flow, weather, and other time series data, successfully predicting water flow for the next six hours [19]. These CNN and LSTM models mostly extract data features from specifically filtered data, ignoring important information that may be contained in the original data. The CLCD model pays special attention to the information that may be filtered out in the original data during processing. By cleverly integrating this ignored information, the CLCD model not only enriches the data features but also enables the model to more comprehensively and deeply understand the inherent laws and structures of the data during the training process.

The above literature review shows that most current research mainly focuses on improving the accuracy and scope of the application of flow meters, but this still cannot meet the actual industrial needs. It is worth noting that in the field of metal solution measurement, no researchers have explored the use of impact force as an auxiliary variable. At the same time, the soft measurement modeling approach based on deep learning models has not yet been applied in the field of metal flow measurement. Therefore, the main contributions of this article are as follows:

(1) To address the problem of the limited measurement of high-temperature molten metal flow, a soft measurement method using impact force as an auxiliary variable is proposed to achieve the online accurate measurement of high-temperature liquid metal flow.
(2) Based on the theoretical foundation of fluid mechanics, a test platform is designed in combination with fluid simulation software to collect impact force sequences under different flow rates.
(3) Using the impact force sequence as input and the flow rate classification level as output, a CLCD deep learning model is constructed, achieving an accuracy rate of 90% with high stability in experimental results.

This article is divided into three chapters in total: The first chapter introduces the project background and significance, points out the defects of the existing high-temperature molten metal flow measurement technologies, and proposes a soft measurement method based on impact force. The second chapter first theoretically explains the relationship between impact force and flow rate, analyzes it using fluid simulation technology, and finally builds an experimental platform to collect data under different flow rates. The third chapter constructs the relationship between impact force and flow rate using the CLCD deep learning model, trains the experimental data, and verifies the effectiveness of the method.

## 2. Theoretical Analysis and Testbed Construction

### 2.1. Simulation Analysis

**Figure 1:**
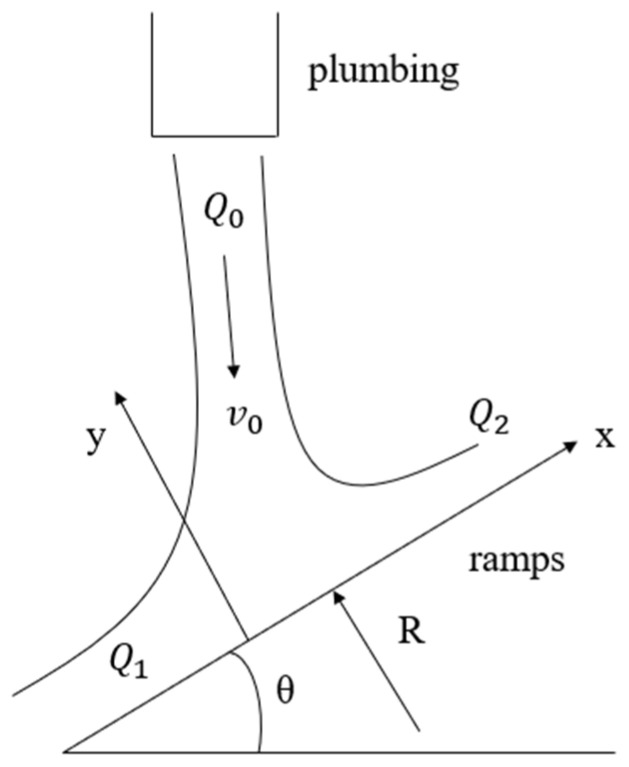
Two-dimensional transient analysis of water impinging on inclined plate.

The water flow impact on the inclined plate for two-dimensional transient analysis, the two-dimensional model is shown in Figure 1. There is a fluid inlet velocity of V_0_, volume flow rate of Q_0_ from the pipeline outflow, and impact on the inclined plate at an angle of θ, and it is then divided into two streams of fluid on the inclined plate, respectively, Q_1_ and Q_2_, according to the theoretical analysis of the impact force R on the flat plate under the ideal conditions, as well as theoretical calculations of the distribution of the flow rate.

The real fluid has a certain viscosity, but for the theoretical analysis here, consider the fluid as an incompressible ideal fluid within the ambient pressure, the air as well as the resistance of the flat plate is not accounted for, and establish a coordinate system as shown in Figure 1; the direction of the impact force on the flat plate is perpendicular to the inclined plane, pointing to the positive direction of the y-axis.

According to the momentum theorem, the increment in the momentum of an object per unit time is equal to the combined force of all the external forces acting on the object, and the momentum equation is listed below:

(1) $$\sum F=\frac{d}{dt}\left(\int_{cv}\rho vdv\right)+\sum {{(q}_{m}v)}_{out}-\sum {{(q}_{m}v)}_{in}$$

where $q_{m}$ is the mass flow rate of outflowing objects per unit time, $v_{out}$ is the velocity of these outflowing substances, and their product represents the momentum outflowing per unit time.

During the shock time, $\frac{d}{dt}(\int_{cv}\rho vdv)$ is zero assuming that the flow is constant. The components of the force F along the x and y directions are given by equations:

(2) $$\sum F_{x}=\sum {{(q}_{m}v_{x})}_{out}-\sum {{(q}_{m}v_{x})}_{in}$$

(3) $$\sum F_{y}=\sum {{(q}_{m}v_{y})}_{out}-\sum {{(q}_{m}v_{y})}_{in}$$

The specific expressions for the combined external forces along x and y can be deduced by combining Figure 1:

(4) $$\sum F_{x}=\left(\rho Q_{1}v_{1}-\rho Q_{2}v_{2}\right)-\rho Q_{0}v_{0}cos\alpha$$

(5) $$\sum F_{y}=0-\rho Q_{0}\left(-v_{0}sin\alpha \right)=\rho Q_{0}v_{0}sin\alpha$$

where $v_{0}$ represents the flow rate when the ramp is not impacted, and $v_{1}$ and $v_{2}$ are the velocities corresponding to the Q_1_ and Q_2_ fluids, respectively.

This ignores viscous friction, so the combined external force is 0 along the x-direction, and the size of the impact force R of the flat plate with a pair of action and reaction forces, so $R=\rho Q_{0}v_{0}sin\alpha$, which is in the opposite direction.

This can be obtained from the continuity equation for flow:

(6) $$Q_{0}=Q_{1}+Q_{2}$$

When a fluid is in the atmosphere, the pressure is equal and ambient everywhere in the fluid.

From the energy equation, we obtain the following:

(7) $$\frac{{v_{0}}^{2}}{2g}=\frac{{v_{1}}^{2}}{2g}=\frac{{v_{2}}^{2}}{2g}$$

where g is the acceleration of gravity

$${so:v}_{0}=v_{1}=v_{2}$$

from $\sum F_{x}=(\rho Q_{1}v_{1}-\rho Q_{2}v_{2})-\rho Q_{0}v_{0}cos\alpha =0$ we obtain the following:

(8) $$Q_{1}-Q_{2}-Q_{0}cos\alpha =0$$

from (6) and (8) we can obtain the following:

(9) $$Q_{1}=\frac{1}{2}Q_{0}\left(1+cos\alpha \right)$$

(10) $$Q_{2}=\frac{1}{2}Q_{0}\left(1-cos\alpha \right)$$

### 2.2. Simulation and Modeling Analysis

**Figure 2:**
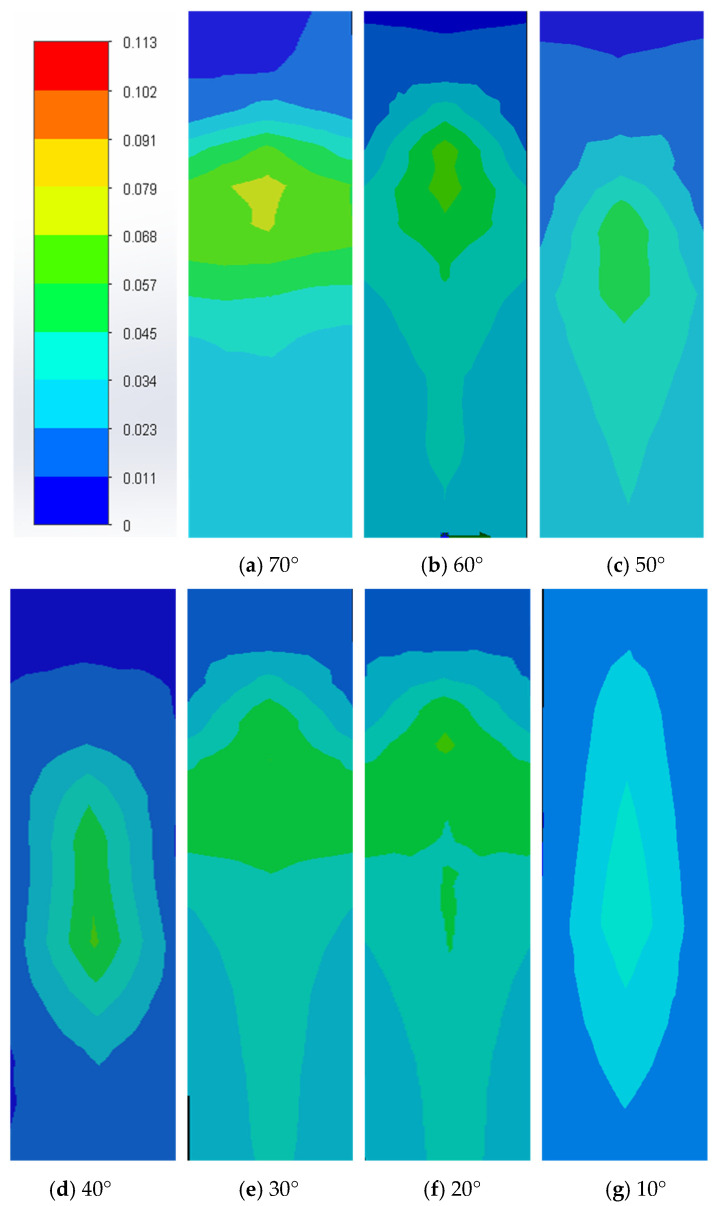
(**a**–**g**) show the simulation of the forces on the inclined plate at different angles of inclination, respectively.

**Figure 3:**
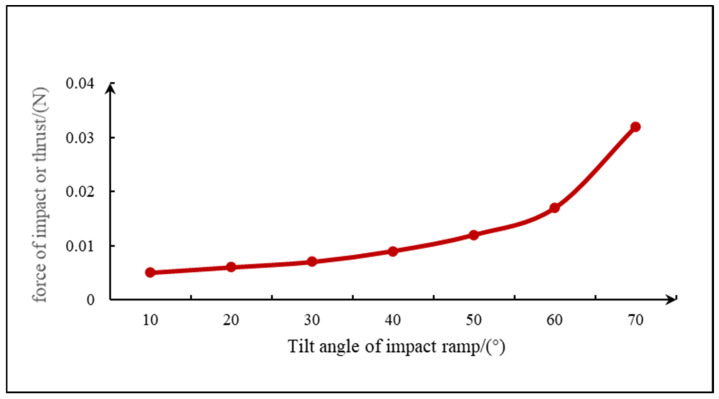
Angle versus impact force graph.

The simulation analysis of the fluid is mainly carried out by using the Flow Simulation function of SolidWorks for fluid analysis. By simulating the flow process of the fluid in the pipeline, the relationship between the inclination angle of the inclined plane and the impact force is studied. In accordance with the basic theory of its impact force and flow rate, $R=\rho Q_{0}v_{0}sin\alpha$, when the density ρ, the incident flow rate $Q_{0}$, and the initial velocity $v_{0}$ are unchanged, the impact force increases with the increase in the inclination angle of the inclined plane. With 0.01 kg/s as the initial conditions of the flow, setting the wall condition adiabatic, choosing level 3 for the mesh level, choosing FFEPlus for the solver, and changing the inclination size in this condition, we change the size of the inclination angle, and the simulation analysis of the different angles of the inclined plane, in the angle of 70° up will be the overall force fluctuations, and the higher the number of degrees, the more it appeared to be unable to calculate the phenomenon. Therefore, when the water impact inclined plane is selected between 10° and 70°, the simulation results of impact inclined plane force are shown in Figure 2. As can be seen from the figure, the force on the inclined plane at an inclination angle of 70° is significantly greater than the other inclined planes. Then, the average impact force is obtained and the line graph with the impact inclination angle is plotted. According to the simulation results, the force rises slowly from 10° to 60° and increases sharply from 60° to 70°, as shown in Figure 3. Therefore, from the simulation calculation and force analysis, 70° is selected as the best tilt angle.

Taking 70° as the inclination angle of the model for analysis, the total duration was set to 3 s, and the data output was recorded at a time step of 0.1 s. To simulate the influence of Earth’s gravity on the research object, we set the gravity components of 0, 0, and −9.81 m/s^2^ in the X, Y, and Z directions, respectively. The internal flow medium was water, and the external medium was air, aiming to comprehensively analyze the fluid dynamic characteristics under both laminar and turbulent flow conditions. The wall conditions were set to be adiabatic with a roughness of 10 μm to better approximate the actual physical environment. The environmental pressure and temperature were set to standard atmospheric conditions, namely atmospheric pressure and room temperature. Meanwhile, to simulate turbulence in the fluid, we set the turbulence intensity to 2%. Regarding mesh division, we adopted a first-level mesh and refined some of the turns to improve mesh quality and reduce simulation errors. The boundary conditions mainly considered the inlet flow rate and outlet pressure. Specifically, the inlet flow rate was set to 0.01 kg/s, and the outlet pressure was maintained at environmental pressure. Through these settings, we were able to simulate the flow process and conduct an in-depth analysis of various parameters.

**Figure 4:**
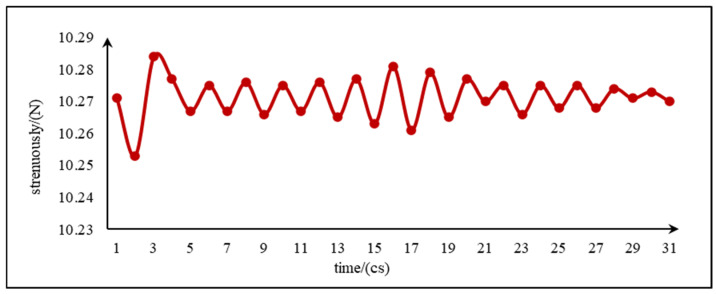
Impact force over time.

**Figure 5:**
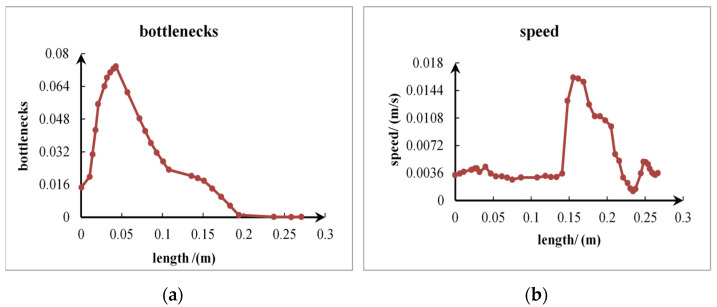
(**a**) represents a plot of axial length vs. number of bottlenecks in the impact ramp, and (**b**) represents a plot of axial length vs. velocity profile in the impact ramp.

**Figure 6:**
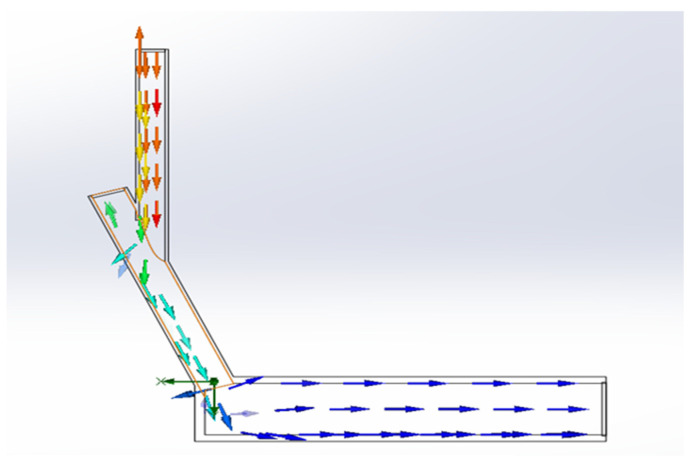
Fluid flow line cross-section.

By simulating the impact process of the fluid on the inclined surface, it can be observed that the impact force on the bottom surface shows a fluctuating trend in a specific range, as shown in Figure 4. And, the analysis of bottleneck number on the centerline of the circular bottom surface is considered as an important dimensionless parameter to measure the strength of the local thermal bottleneck. The presence of the bottleneck number implies that the fluid flow rate, flow, or pressure is significantly restricted at these particular locations, which in turn has a profound effect on the overall hydrodynamic process. In order to obtain the point of maximum impact, the point of maximum flow velocity is selected and the limitation of the number of bottlenecks at that location is also taken into account, as can be seen in Figure 5, where the velocity profile shows that the fluid velocity reaches its peak at about 0.16 m, and in comparison the number of bottlenecks in this region is observed to be relatively small. In addition, in order to understand the trend in the fluid flow, the flow profile of the fluid is plotted as shown in Figure 6, with the arrows representing the direction of the flow from which the location of the drop point of the water flow in the pipe can be seen. Based on the above simulation results, the best location for sensor installation was deduced, thus optimizing the overall design structure and laying a solid foundation for the subsequent impact force acquisition.

#### 2.2.1. Structural Design of Experimental Platform

**Figure 7:**
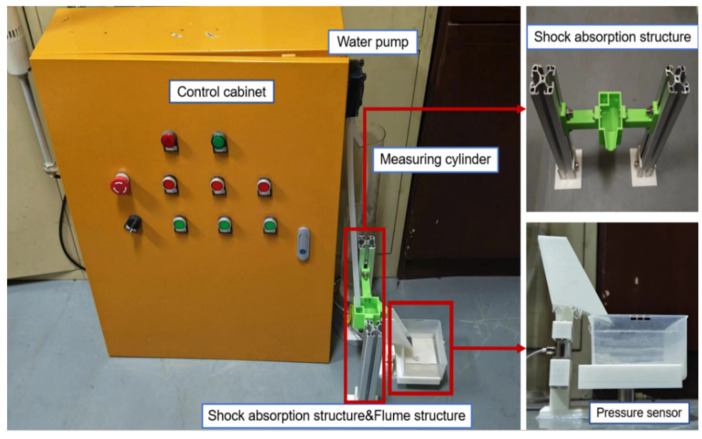
Test environment setup and impact part diagram.

In order to collect the impact force and flow data and verify the feasibility of using impact force as an auxiliary variable to measure as proposed in the paper, water is used as the fluid medium to build an experimental platform for predicting the flow based on impact force. As shown in Figure 7, the platform is mainly composed of core components such as a control system, pressure sensor, and data acquisition software platform. By changing the key parameters such as the rotational speed, acceleration, and running time of the pump, the acquisition of impact force data under different flow rates is realized.

The design of the impact force component of the entire experimental platform is crucial to the accuracy of data collection, which mainly consists of two parts: shock absorption structure and water tank structure. The core of the shock absorption structure design lies in introducing the aluminum profile water storage tank structure as a pre-water-flow channel, whose main function is to isolate the direct contact between the hose of the peristaltic pump and the water tank structure. This successfully reduces the potential impact of the vibrations generated during pump operation on the water tank structure, thereby significantly improving the stability and accuracy of sensor data collection. The water tank structure is equipped with two sensors which are installed at the bottom of the water tank and the plastic box, respectively. The pressure sensor at the bottom of the water tank is used to accurately measure the impact force generated when the solution impacts the bottom of the water tank, while the sensor at the bottom of the plastic box is used to measure the total mass accumulated by each discharge of the pump, which serves as a classification label.

During the overall construction of the test platform, the peristaltic action of the pump is mainly executed precisely by the control system. The key hardware devices and models used in the control system, as well as the corresponding parameters and attributes are listed in Table 1 below. 

**Figure 8:**
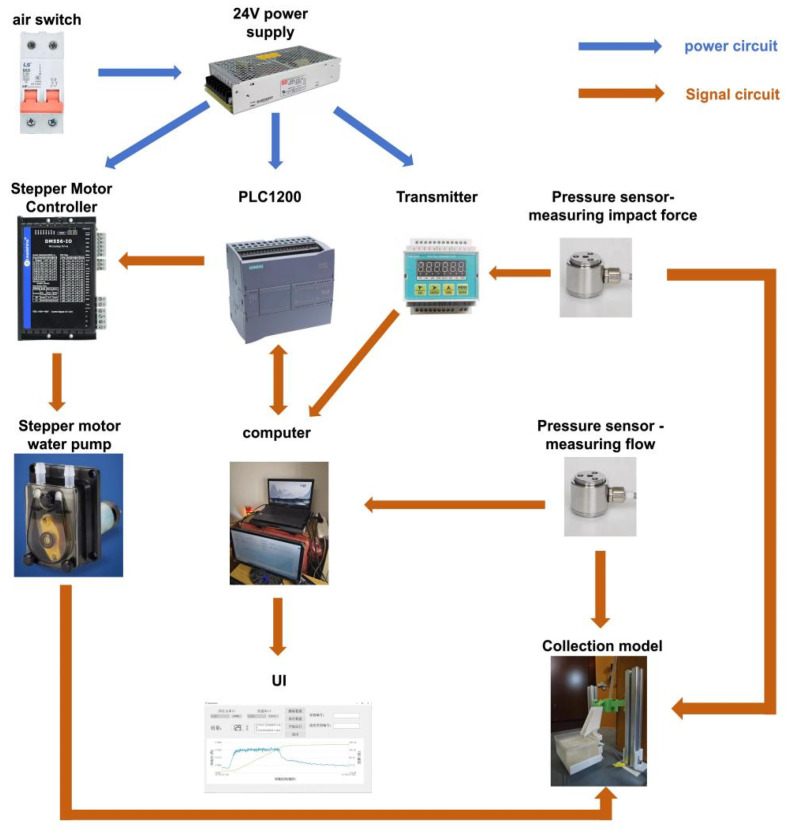
Control system hardware design diagram.

The block diagram of the hardware control system of the whole data acquisition system is shown in Figure 8. The control system contains two major parts: data acquisition and motor control. PLC connects with the stepper motor controller and outputs the PTO signal so as to drive the stepper motor pump movement and feedback the completion signal to the computer. Through network communication, the computer establishes communication with the PLC to control the stepper motor. The pressure sensor value is read through 232 communication.

#### 2.2.2. Software Design of Acquisition System

**Figure 9:**
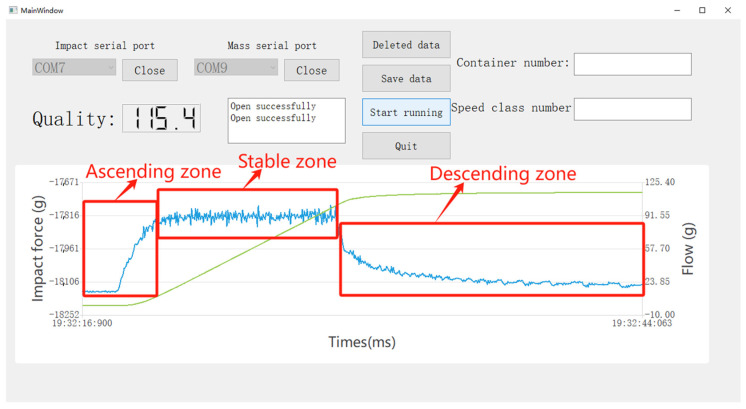
Acquisition system software interface design.

The software interface of the data acquisition system is shown in Figure 9, which is mainly composed of four functional modules: communication setting, control parameters, saving parameters, and data display.

The blue curve in the figure depicts the trend in impact force over time, while the green curve reflects the change in flow rate over time. The trend in the impact force over time can be divided into three phases: an initial rise phase, a fluctuating vibration phase, and a subsequent fall phase. In the initial rise phase, the pump started and gradually accelerated to a predetermined speed, at which point the pump began to extract water, resulting in a gradual increase in shock force. Subsequently, it entered the fluctuating vibration stage, the pump speed stabilized, and pumping water continued to impact the bottom of the tank, resulting in vibration effects, and the impact force showed a fluctuation state. Eventually, with the gradual reduction in the pump speed until it stopped, the amount of water pumped out to reduce the impact gradually returned to the initial state, the formation of the decline stage.

In the rising stage of the blue curve, as the pump has just begun to run, there is no water flow, so the flow rate at this time is 0. With the blue curve into the vibration stage, the pump begins to stabilize the output of water flow, and the flow rate gradually increases. When the blue curve is in the falling stage, the flow rate reaches its maximum value and then stabilizes, indicating that the pumping process has been basically completed.

**Figure 10:**
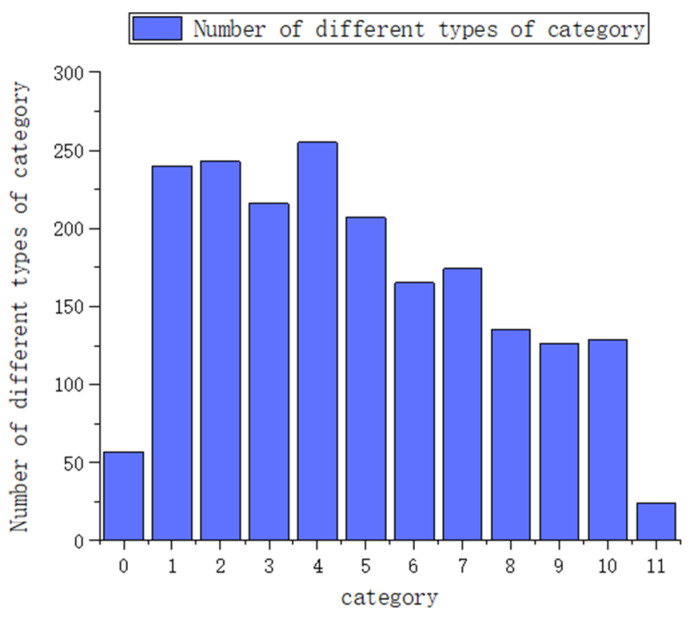
Statistical chart of classification category data.

The experimental data collection mainly collects the time series impact force data as well as the total mass of flow for each total time series, in which the sampling frequency is 300 Hz. The categorization labels are mainly based on the total mass of water flow at each time. The total mass below 50 g is category 0, above 150 g is category 11, in the middle of 50 g to 150 g, from 50 g to 60 g is category 1, and with every increase of 10 g, the number of categories increases by 1. In total, there are 12 categories, and the number of samples corresponding to each category is shown in Figure 10.

## 3. Analysis of Test Results

Firstly, the simulation experiment platform is built, the frequency and time of the pump are adjusted, and the time series data of the water flow impact force is obtained through the pressure sensor. Subsequently, the impact force sequence is processed using the sliding window filtering technique to filter out the noise interference and make the data fluctuations stable and smooth while accelerating the data processing speed. In order to ensure the robustness of the model, adapt to the changes under different experimental conditions, and better adapt to the input requirements of the model, the WRP algorithm is used to normalize the length of the data, and the time series of the original length is cut into multiple copies of the data, each of which generates the elements of a new one of the time series according to the weight function.

Where the weight function f(x) is shown below:

$$f\left(x\right)=\left\{\begin{array}{c} 0.1\left|x^{3}\right|-0.5x^{2}+0.3\left|x\right|+0.9\;\;\;\;1\leq \;\left|x\right|\leq 3\\ -0.2x^{2}+1\;\;\;\;\;\;\;\;\;\;\;\;\;\;\;\;\;\;\;\;\;\;\;\;\;\;\;\;\;\;\;\;\;\;\;\;\;\;\;-1<x<1\\ 0\;\;\;\;\;\;\;\;\;\;\;\;\;\;\;\;\;\;\;\;\;\;\;\;\;\;\;\;\;\;\;\;\;\;\;\;\;\;\;\;\;\;\;\;\;\;\;\;\;\;\;\;\;\;\;\;\;\;\;\;\;\;\;\;\;\;\;\;\;\;\left|x\right|>3 \end{array}\right.$$

**Figure 11:**
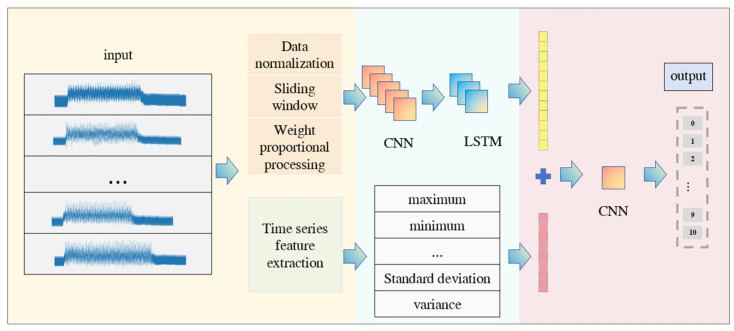
CLCD model framework based on impact force.

The processed data are divided into a training set and test set in a 4:1 ratio, and input into the CNN-LSTM model for pre-training, and at the same time, the time series feature values of the original time series impact data are extracted, including maximum, minimum, mean, median, skewness, kurtosis, standard deviation, and variance. The output of the fully connected layer in the pre-training as well as the time series feature values are achieved through data splicing and stored in the feature layer of the network, which fuses the information from different sources, thus improving the generalization ability of the model. Finally, the feature layer is trained based on CNN to output the total traffic classification class. The whole process is shown in Figure 11.

**Figure 12:**
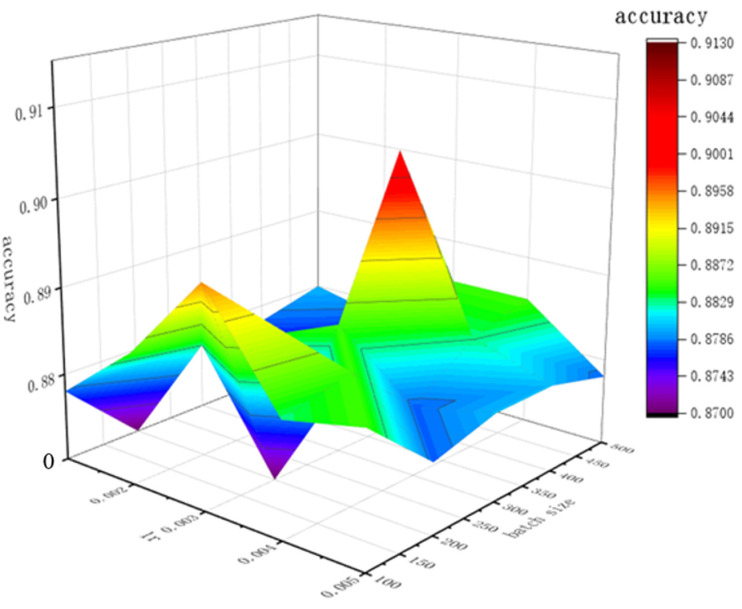
Plot of training accuracy results for different learning rates and number of training sessions in the CLCD model.

The CLCD model is not only capable of extracting features in the time domain and capturing the before-and-after temporal relationship, but also extracting time series eigenvalues of the original data, providing more information for the model, improving the model’s ability to understand the change in the impact force, integrating information from different sources, and improving the generalization ability so that it can accurately understand and classify the complex dynamic change in the impact force of the water flow under different frequency and time conditions. However, different parameters such as learning rate and the number of training times have a large impact on the classification accuracy of the CLCD model. According to Figure 12, it can be seen that the maximum value is reached when the learning rate is 0.003 and the batch size is 400.

**Figure 13:**
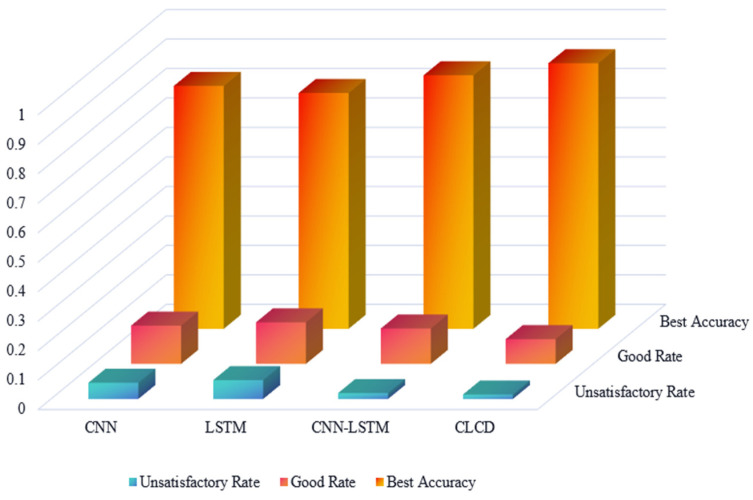
CNN, LSTM, CNN-LSTM, and CLCD classification result evaluation.

The CLCD model was trained with CNN, LSTM, and CNN-LSTM, and the results were evaluated in a comparative analysis. The excellent rate, good rate, and failure rate are used to represent the probability that the model training classification results are completely accurate, the probability that the classification error difference is within two classes, and the probability that the classification class is greater than two classes, respectively. The results are shown in Figure 13 below.

CNN extracts the local features of the input data through the sliding operations of the convolutional kernel, and its advantage is that it can effectively capture the hierarchical features of the input data, and gradually extract the abstract and high-level features of the data through multiple convolutional and pooling layers, which is able to learn the fluctuation of the water impact force as well as the other feature changes in the processing of the water impact force data. The LSTM, through the gating mechanism, is able to learn inside the network and maintain long-term temporal dependencies, which is very critical for the time series data of water flow impact force, and is able to address the fact that the impact force may be affected by previous time points and there are long term dynamic patterns. So, it is well evident from the figure that the results of the individual model training of CNN and LSTM are not bad, and that the accuracy as well as the excellence rate of CNN-LSTM is higher than the effect of individual class prediction of CNN and LSTM.

However, the CLCD model incorporates the original data time series eigenvalues, and compared with the CNN-LSTM model, it makes up for the neglect of the time series information in the original data, helps to enrich the data characterization, and can accurately understand and categorize the complex dynamic changes in the water flow impact force under different frequency and time conditions, and it can be clearly seen through the experimental results that the CLCD model’s indexes are better than the other models and the classification effect is better.

**Figure 14:**
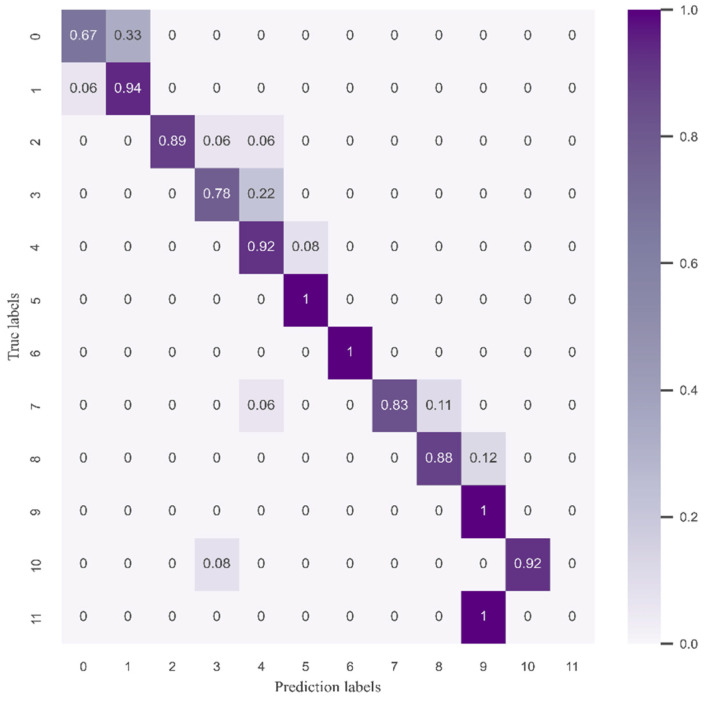
Diagram of the CLCD model training confusion matrix.

In order to better analyze the training classification results of the CLCD model, the confusion matrix is plotted as in Figure 14. More data were collected by adjusting the different speeds and running times of the pumps, but the small number of samples with a mass of more than 150 g led to the unsatisfactory performance of category 11 in terms of classification accuracy in the test dataset, which even reached 0%. However, it should be emphasized that despite the low accuracy of category 11, its error level is still effectively controlled within two levels, and thus still considered as an acceptable classification range. In addition, considering that in practical engineering applications, the flow rate is mainly concentrated between 60 g and 120 g, the accuracy of the CLCD model in this interval performs particularly well and fully meets the practical needs.

**Figure 15:**
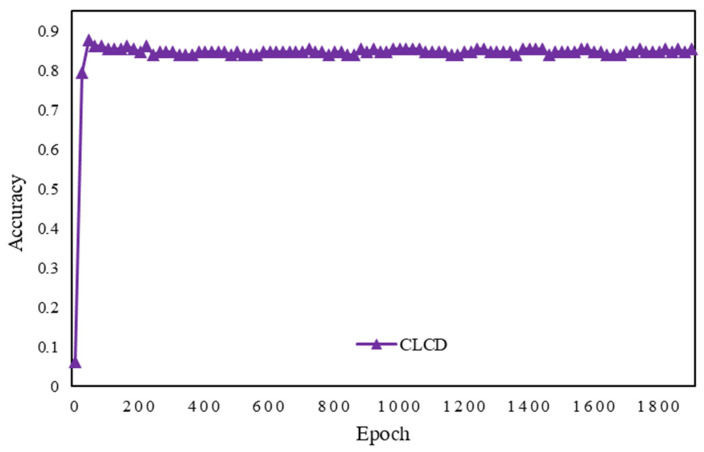
Epoch vs. accuracy plot for CLCD.

**Figure 16:**
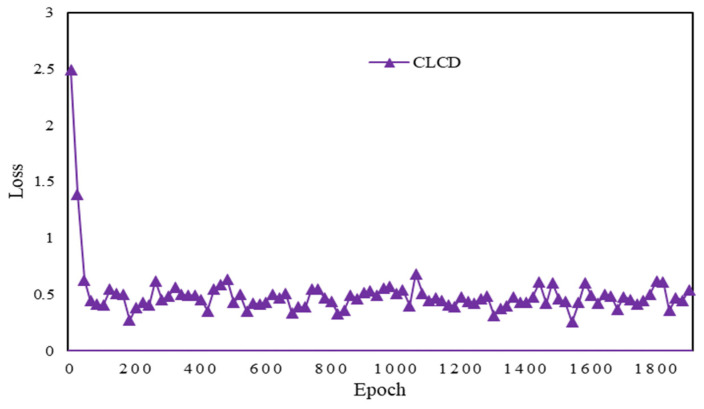
Epoch vs. Loss plot for CLCD.

After training through the CLCD model, its accuracy and loss rate graphs are shown below in Figure 15 and Figure 16, with the increase in the number of training generation’s accuracy instantly reaching the highest value, and then gradually smoothing; the loss rate is also at the beginning of the drop below 0.5, and then fluctuates around 0.3.

**Figure 17:**
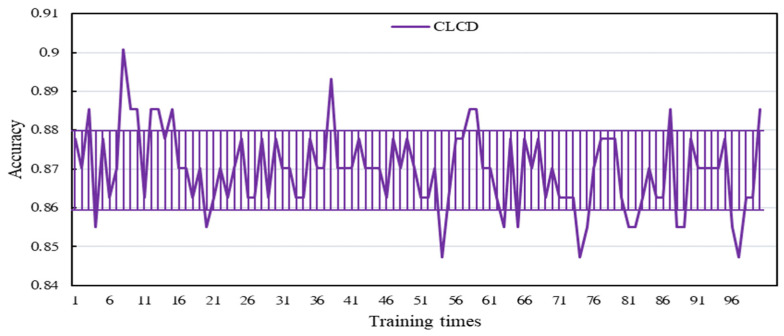
Accuracy of CLCD model training results vs. number of training sessions.

In order to verify the stability of the flow classification prediction, 100 flow classification predictions were made for the model, and the accuracy of each classification was recorded in detail. As shown in Figure 17, after statistical analysis, the accuracy error line was plotted, and its fluctuation range was stable between 0.86 and 0.87, showing that the model is highly stable.

## 4. Conclusions

In this paper, to solve problems such as unstable casting quality caused by the difficulty of controlling the flow in the casting process of high-temperature metal solution, according to the actual industrial environment, we propose to analyze and experimentally validate the soft measurement method for the flow process of high-temperature metal solution by taking the impact force as the observation variable to realize the online measurement of the flow. The CLCD deep learning model, which is mainly based on data, is adopted, and based on the fluid mechanics analysis and simulation results, the test platform is built to obtain the sample set of impact force sequences with different flow rates, and the model is trained with different flow size levels as output, with an accuracy rate of 90% and high stability. Due to the limitations of the test environment, room temperature water is used as an alternative to start the acquisition of the impact force training set, which is quite different from the actual metal solution in terms of density, fluidity, viscosity, and many other parameters, but the density of the liquid metal is usually several times that of the water, which means that the liquid metal has a greater mass under the same volume, and theoretically, it will also produce a greater impact force, and in terms of viscosity, although the viscosity of the liquid metal is 0.0022 kg/(m-s), which is slightly higher than the viscosity of water, it has good fluidity, so further test studies will be carried out subsequently for different metal solutions to verify the effectiveness of the method in this paper.

## Figures and Tables

**Table 1:** Hardware of the control system.

Equipment Name	Model	Parameters and Properties
PLC (SIEMENS, Beijing, China)	PLC1200 1214C DC/DC/DC	14 digital input pins 10 digital output pins 2 analog input pins 4 PTO pulse output channels
Peristaltic Pump (JIHPUMP, Chongqing, China)	JIHPUMP 304K	Maximum flow rate 3000 mL/min Pump tube inner diameter 7.8 mm Pump tube outer diameter 11 mm
Stepper Motor Controller (Leadshine, Shenzhen, China)	Leadshine DM556	
Air Switch (LS, Zhejiang, China)	LS BKN Series	Rated current 1-63A
Pressure Sensors (ARIZON, Jiangsu, China)	AR-DN333	Accuracy 0.1% Measurement range 10/20/30/50/100/200/500/1000 N

## Data Availability

The data supporting this study’s findings are available from the corresponding author upon reasonable request.
